# Comorbidity burden of patients with Parkinson’s disease and Parkinsonism between 2003 and 2012: A multicentre, nationwide, retrospective study in China

**DOI:** 10.1038/s41598-017-01795-0

**Published:** 2017-05-10

**Authors:** Xin Wang, Fan Zeng, Wang-Sheng Jin, Chi Zhu, Qing-Hua Wang, Xian-Le Bu, Hong-Bo Luo, Hai-Qiang Zou, Jie Pu, Zhong-He Zhou, Xiao-Ping Cui, Qing-Song Wang, Xiang-Qun Shi, Wei Han, Qiang Wu, Hui-Sheng Chen, Hang Lin, Li-Li Zhang, Meng Zhang, Yan Lian, Zhi-Qiang Xu, Hua-Dong Zhou, Tao Zhang, Yan-Jiang Wang

**Affiliations:** 1 Department of Neurology and Center for Clinical Neuroscience, Daping Hospital, Third Military Medical University, Chongqing, 400042 China; 2 Department of Neurology, Lanzhou General Hospital, Lanzhou, 730050 China; 3 Department of Neurology, Guangzhou General Hospital, Guangzhou, 510010 China; 4 Department of Neurology, Wuhan General Hospital, Wuhan, 430070 China; 5 Department of Neurology, Shenyang General Hospital, Shenyang, 110016 China; 6 Department of Neurology, Fuzhou General Hospital, Fuzhou, 350025 China; 7 Department of Neurology, Chengdu General Hospital, Chengdu, 610083 China; 8 Department of Prevention Medicine, Guangzhou General Hospital, Guangzhou, 510010 China; 9 Department of Preventive Medicine, Daping Hospital, Third Military Medical University, Chongqing, 400042 China

## Abstract

Parkinson’s disease (PD) and Parkinsonism are common neurodegenerative disorders with continuously increasing prevalence, causing high global burdens. However, data concerning the comorbidity burden of patients with PD or Parkinsonism in China are lacking. To investigate the health condition and comorbidity burden, a total of 3367 PD and 823 Parkinsonism patients were included from seven tertiary hospitals in seven cities across China from 2003 to 2012. Their comorbidity burden was collected and quantified by the Elixhauser Comorbidity Index (ECI) and Charlson Comorbidity Index (CCI). The comorbidity spectra differed between PD and Parkinsonism patients. Compared with PD patients, Parkinsonism patients were older (69.8 ± 11.5 vs. 67.9 ± 11.4, *P* < 0.001); had a higher comorbidity burden, including ECI (1.1 ± 1.2 vs. 1.0 ± 1.2, *P* < 0.001) and CCI (1.3 ± 1.6 vs. 1.1 ± 1.5, *P* < 0.001); and had higher hospitalization expenses. The ECI (1.1 ± 1.3 vs. 0.9 ± 1.1, *P* < 0.001) and CCI (1.3 ± 1.6 vs. 0.9 ± 1.2, *P* < 0.001) were higher in males than in females. The average length of stay and daily hospitalization expenses increased with age, as did ECI and CCI. This is the first study to report the disease burden of Chinese PD and Parkinsonism patients. It provides useful information to better understand their health status, and to raise the awareness of clinicians for providing better health care.

## Introduction

With the increase in the worldwide life expectancy, the chronic disease burden has been growing^1^. Such a trend is likely to continue, especially in developing countries^2, 3^. Studies on disease burden are critical to guiding health care planning and social and economic policy^1^. Parkinson’s disease (PD) and secondary Parkinsonism (Parkinsonism) are two common chronic neurologic disorders that usually affect the elderly population with continuously increasing prevalence, and they have caused a heavy global financial burden^4^. However, the general health status of patients with PD and Parkinsonism in China remains largely unknown.

Previous studies showed that the prevalence rates of dementia and depression in PD ranges from 17.4% to 31.5% and 2.7% to over 90%, respectively^5, 6^. In a large epidemiological survey estimating the hospitalization burden of 1765 primary PD patients in Spain, the most frequent comorbidities were unspecified essential hypertension (34%), diabetes mellitus type II (15%), unspecified hyperlipidaemia (14%), depressive disorder (8%), atrial fibrillation (7%), and urinary tract infection site not specified (7%)^7^. Another study from the Canadian Community Health Survey reported that back problems (36.0%), arthritis (34.3%), hypertension (29.3%), cataracts (23.9%) and urinary incontinence (11.3%) were the most common comorbidities in PD patients^8^. With the largest population in the world, China is facing population ageing to the extent that people aged 60 years or older account for 13.3% of the total population^9^. However, data on the comorbidity burden of patients with PD or Parkinsonism in China are lacking at the national level. This nationwide, multicentre, hospital-based retrospective study was conducted to investigate the general health condition, especially comorbidity burden, of PD and Parkinsonism patients in China.

## Results

### Characteristics of study subjects

A total of 4,190 inpatients, including 3,367 patients with PD and 823 patients with Parkinsonism, were included in our study. PD patients were more frequently admitted than Parkinsonism patients (80.4% vs. 19.6%). The mean age of all patients was 68.3 ± 11.4 years, and the mean age of the Parkinsonism patients was older than PD patients (69.8 ± 11.5 vs. 67.9 ± 11.4, *P* < 0.001). There was a male predominance (61.0% male vs. 39.0% female) in our study. No difference in gender composition between PD and Parkinsonism patients was observed.

The Elixhauser Comorbidity Index (ECI) and Charlson Comorbidity Index (CCI) are presented in Table 1. For all patients, the ECI was 1.0 ± 1.2 and CCI was 1.1 ± 1.5. Compared to the patients with PD, the Parkinsonism patients had a higher ECI (1.1 ± 1.2 vs. 1.0 ± 1.2, *P* < 0.001) and CCI (1.3 ± 1.6 vs. 1.1 ± 1.5, *P* < 0.001). After adjusting for the confounding effect of age, only the difference in CCI remained statistically significant (*P* = 0.004). The most frequent comorbidities for the PD patients were cerebrovascular disease (42.53%), hypertension (33.17%), diabetes (10.60%), chronic pulmonary disease (6.98%) and paralysis (5.53%). For the Parkinsonism patients, cerebrovascular disease (53.22%), hypertension (39.00%), diabetes (11.66%), paralysis (11.06%) and dementia (7.05%) were more common. Parkinsonism patients more frequently had cerebrovascular disease, dementia, paralysis, hypertension, weight loss, and drug abuse than patients with PD, but they had a lower prevalence of solid tumor without metastasis and mild liver disease (see Supplementary Table S1). These results indicated that the comorbidity burden is relatively heavier in Parkinsonism patients than in patients with PD.

**Table 1:** Characteristics of study subjects.

	Overall (4,190)	PD (3,367)	Parkinsonism (823)		*P* value
Gender (n, %)				χ^2^ = 0.299	0.585
Male	2,555 (61.0)	2,060 (61.2)	495 (60.1)		
Female	1,635 (39.0)	1,307 (38.8)	328 (39.9)		
Age (mean, SD; y)	68.3 (11.4)	67.9 (11.4)	69.8 (11.5)	t = 4.258	**<0.001**
<50	279	234 (6.9)	45 (5.5)	χ^2^ = 25.946	**<0.001**
50–59	628	536 (15.9)	92 (11.2)		
60–69	1,132	929 (27.6)	203 (24.7)		
70–79	1476	1,153 (34.2)	323 (39.2)		
80+	675	515 (15.3)	160 (19.4)		
ECI (mean, SD)	1.0 (1.2)	1.0 (1.2)	1.1 (1.2)	t = 2.613	**<0.001**
0	1849	1,507 (44.8)	342 (41.6)	χ^2^ = 12.959	**0.024**
1	1,201	980 (29.1)	221 (26.9)		**0.040** ^**a**^
2	689	538 (16.0)	151 (18.3)		
3	258	192 (5.7)	66 (8.0)		
4	130	98 (2.9)	32 (3.9)		
5+	63	52 (1.5)	11 (1.3)		
CCI (mean, SD)	1.1 (1.5)	1.1 (1.5)	1.3 (1.6)	t = 3.450	**<0.001**
0	1,746	1,433 (42.6)	313 (38.0)	χ^2^ = 28.929	**<0.001**
1	1,361	1,110 (33.0)	251 (30.5)		0.251^a^
2	513	405 (12.0)	108 (13.1)		
3	271	191 (5.7)	80 (9.7)		
4	136	97 (2.9)	39 (4.7)		
5+	163	131 (3.9)	32 (3.9)		

^a^ *P* values after adjustment for the influence of age on the comorbidity burden with the general linear model.

### Comorbidity burden and age

**Figure 1:**
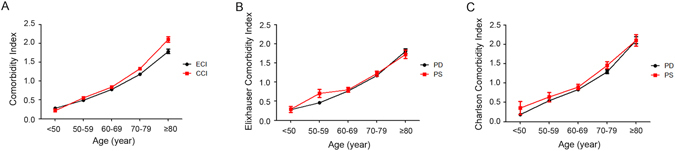
Associations of the comorbidity burden with age. All subjects were divided into 5 sub-groups (<50 years, 50–59 years, 60–69 years, 70–79 years and ≥80 years) by age. The comorbidity indices, including the Comorbidity Index (**A**), Elixhauser Comorbidity Index (**B**) and Charlson Cormorbidity Index (**C**), increased with age in both patients with PD and Parkinsonism.

ECI and CCI both increased with age in all patients (ECI Spearman r = 0.371, *P* < 0.001 and CCI Spearman r = 0.398, *P* < 0.001) (Fig. 1A), PD patients (ECI Spearman r = 0.384, *P* < 0.001 and CCI Spearman r = 0.403, *P* < 0.001) and Parkinsonism patients (ECI Spearman r = 0.311, *P* < 0.001 and CCI Spearman r = 0.364, *P* < 0.001) (Fig. 1B,C). Compared with PD patients, Parkinsonism patients had a higher ECI and CCI in almost all age subgroups. The prevalence of each comorbidity also increased with age in both PD and Parkinsonism patients (see Supplementary Tables S2, S3 and S4).

### Comorbidity burden and gender

In all patients, males had a higher ECI (1.1 ± 1.3 vs. 0.9 ± 1.1, *P* < 0.001) and CCI (1.3 ± 1.6 vs. 0.9 ± 1.2, *P* < 0.001) than females. For both PD and Parkinsonism patients, males also had a higher ECI (1.1 ± 1.3 vs. 0.9 ± 1.1, *P* < 0.001 and 1.2 ± 1.3 vs. 0.9 ± 1.1, *P* = 0.029) and CCI (1.2 ± 1.6 vs. 0.9 ± 1.2, *P* < 0.001 and 1.5 ± 1.7 vs. 1.1 ± 1.3, *P* = 0.002). Males more frequently suffered from comorbidities, including myocardial infarction, cerebrovascular disease, peptic ulcer disease, liver diseases, dementia, paralysis, renal failure, cancer, cardiac arrhythmia, hypertension, and alcohol abuse. Connective tissue diseases and psychoses were more common in females.

### Average length of stay and daily hospitalization expense

Males had higher daily hospitalization expenses and longer hospital stays than females. The average length of stay (ALS) and daily hospitalization expense increased with an increased ECI or CCI (Table 2) in both PD and Parkinsonism patients (see Supplementary Tables S5 and S6). The ALS and daily hospitalization expense also increased with age (Table 2) in both PD and Parkinsonism patients (see Supplementary Tables S5 and S6).

**Table 2:** Length of stay and hospitalization expense for all patients.

Overall	Length of Stay (days)			Cost per day^a^		
	Mean (SD)		*P* value	Mean (SD)		*P* value
Sex		Z = −4.194	<0.001		Z = −4.149	<0.001
Male	16.8 (16.2)			1.10 (1.01)		
Female	13.7 (12.4)			0.96 (0.74)		
Age		χ^2^ = 133.201	<0.001		χ^2^ = 28.035	<0.001
<50	11.8 (11.2)			0.80 (0.43)		
50–59	10.6 (6.6)			0.95 (0.69)		
60–69	13.6 (14.8)			1.02 (1.23)		
70–79	16.3 (14.1)			1.09 (0.78)		
≥80	23.3 (20.0)			1.14 (0.89)		
ECI		χ^2^ = 92.168	<0.001		χ^2^ = 60.660	<0.001
0	13.1 (16.0)			0.91 (1.04)		
1	16.0 (15.1)			1.03 (0.75)		
2	16.3 (12.8)			1.16 (0.89)		
3	19.4 (14.7)			1.22 (1.17)		
4	19.0 (13.1)			1.41 (0.85)		
5+	26.2 (12.7)			1.22 (0.61)		
CCI		χ^2^ = 108.677	<0.001		χ^2^ = 77.517	<0.001
0	14.1 (15.7)			0.87 (0.65)		
1	14.0 (14.1)			1.01 (1.00)		
2	15.9 (12.9)			1.22 (0.87)		
3	19.8 (14.9)			1.32 (1.02)		
4	24.8 (22.7)			1.33 (1.64)		
5+	24.9 (12.7)			1.17 (0.70)		

^a^Each value was divided by 1057 for standardization.

## Discussion

This is the first nationwide study exploring the comorbidity burden of patients with PD and Parkinsonism in China. To better represent the Chinese population, a large cohort of 4,190 patients from seven tertiary hospitals in seven cities located in different regions of China, for a period of ten years, was included in this study. The results demonstrated the heavy comorbidity burden and medical expense in patients with PD and Parkinsonism.

Overall, compared with PD patients, Parkinsonism patients had a heavier comorbidity burden, and their hospitalization expenses were significantly higher. Therefore, Parkinsonism patients were presumed to be in worse condition than PD patients. Both ECI and CCI increased with age. Moreover, after multivariate analysis to adjust for the confounding effect of age, the difference in CCI (but not ECI) remained statistically significant, suggesting that age impacts the comorbidity burden.

We found that PD was consistently more frequent in males than in females with an approximate ratio of 1:1.6. This finding is consistent with the previously reported ratio of 1:1.5 in Western countries^10^. The reason for this increased risk in males remained unclear. A previous study suggested that male patients may more often suffer from head trauma and toxicant exposure, which are risk factors for PD^11^. Another possible reason is that oestrogen may be neuroprotective^12^. ECI and CCI are also higher in male patients than female patients. This may be because males had a higher prevalence of common diseases^2^. Accordingly, the ALS and daily hospital expenses were higher for the males than for females. Differences in the comorbidity spectrum between males and females were also revealed in our study, as previously reported^13^. The prevalence of connective tissue diseases and psychoses was lower in males. Conversely, the prevalence of myocardial infarction, cerebrovascular disease, chronic pulmonary disease, peptic ulcer disease, liver disease, dementia, paralysis, renal failure, cancer (including solid tumor without metastasis and metastatic cancer), cardiac arrhythmia, hypertension, and alcohol abuse is higher in males than in females.

The patients with PD and Parkinsonism had the same tendency in the ECI and CCI, respectively. A systematic analysis for the global burden of disease showed that despite continuous increases in the global life expectancy over the past 40 years for both men and women, the accompanying disease burden has continuously become heavier^14^. This upward tendency might be due to more advanced technologies for the acute diagnosis of patients, revealing more disease information.

Among patients with PD or Parkinsonism who are usually treated with dopaminergic drugs, abnormalities in blood pressure are commonly seen. In our entire cohort, 1,438 patients (34.3%) were diagnosed with hypertension; the prevalence rates of hypertension among individuals aged 60–69, 70–79, and 80 years or older were 28.9%, 42.4% and 53.6%, respectively. These frequencies are lower than those reported for the general population of 57.1% in people aged 55–64 years, 68.6% in people aged 65–74 years and 72.8% in people aged 75 years or older^15^. The prevalence of hypertension among adults aged 65 years and older was 70.8% in the United States^16^. The negative correlation between hypertension and PD or Parkinsonism in the study indicated that the abnormalities in blood pressure are not a direct result of the effects of these drugs; instead, they are possibly due to sympathetic noradrenergic denervation and baroreflex failure in patients with PD or Parkinsonism, as suggested in previous studies^17^. In addition, cardiac sympathetic noradrenergic denervation also contributes to the lower frequency of hypertension in patients with PD^17^.

Dementia occurs as part of the disease process, which directly leads to a decrease in the quality of life for PD and Parkinsonism patients. The prevalence of dementia in our cohort is 4.9% for all patients and 6.9% for patients aged 65 years and older, which is considerably higher than the prevalence of 5.14% observed in the general Chinese population aged 65 years and older^18^. A systematic review showed that the prevalence of dementia in patients with PD ranges from 17.4% to 31.5%, with an average of 24.5%^5^, which is higher than that in our cohort.

Previous studies have suggested that symptoms of depression might develop, even in the pre-motor stage of PD, and might thus be evident in individuals at the time of diagnosis^19^. The underlying mechanisms of depression in PD remain poorly understood, but changes in the brain structure, signalling by neurotransmitters, and levels of inflammatory and neurotrophic factors might be involved in the pathogenesis of depression in PD patients^19^. The prevalence of depression among PD patients has been found to range from 2.7% to more than 90%, with a mean of 17%^6^. In our study, only 1.2% of the participants were diagnosed with depression. It is possible that depression might be underestimated by doctors when they mainly focus on treating the motor symptoms of PD or Parkinsonism patients.

The overall prevalence of diabetes mellitus (DM) was estimated to be 11.6% in the Chinese population, which was 22.5% for people aged 60–69 years and 23.5% for people aged 70 years or older^20^. The prevalence of DM among United States adults aged 65 years and older was found to be 21.2%^16^. The prevalence of DM in our study was 12.3% overall, 9.7% for people 60–69 years of age and 16.6% for people 70 years of age or older, suggesting a tendency of a lower DM prevalence in patients with PD or Parkinsonism. Previous studies on the association between DM and PD or Parkinsonism demonstrated conflicting results^21, 22^. Fewer vascular risk factors shared by PD and Parkinsonism patients compared with the general population may be a major contributor to their lower prevalence of DM^21^. However, whether the prevalence of DM is lower in PD patients requires further investigation.

One strength of this study is the large sample size of patients with PD and Parkinsonism from seven cities in different regions across China from 2003 to 2012, which might be representative of the Chinese PD and Parkinsonism population. The main limitation is that, as a retrospective study, cluster random selection for recruiting hospital or stratified sampling was not applied, and only the patients admitted to the hospital were enrolled. These patients would have more severe PD or Parkinsonism and thus more comorbidities. This limitation might make selection bias, such as Berkson’s bias, unavoidable. Additionally, inter-rater agreement could not be evaluated in this retrospective study. Therefore, the results should be interpreted as a measure of the comorbidities and hospital burden in patients with PD and Parkinsonism rather than as the actual incidence or prevalence rates in general patients with PD and Parkinsonism. Additionally, due to the nature of this study, a sex- and age-matched control from the general population was not enrolled, making it difficult to provide a fair picture of the comorbidity burden in general patients with PD and Parkinsonism. Another limitation was that Parkinsonian conditions are notoriously difficult to accurately diagnose, even for movement disorder specialists^23^. Although tertiary hospitals with better accuracy in disease diagnosis and established computing systems for the medical record were selected in our study, it is possible that the hospital-based case ascertainment method used in our study may not allow for a very accurate inter-group comparison between individuals with PD and Parkinsonism.

In conclusion, this is the first study to depict the disease burden of a large cohort of Chinese patients with PD and Parkinsonism nationwide. The results offer a better understanding of the overall health status of patients with PD or Parkinsonism and provide a reference for future studies and assist in comparisons with patients who have other types of neurologic diseases. Moreover, this study is expected to raise clinician awareness and help clinicians provide better health care to patients.

## Methods

### Study subjects

To obtain comorbidities in patients with PD or Parkinsonism in China, a hospital-based retrospective survey was performed in seven cities in China: Chongqing (southwest China), Chengdu (southwest China), Guangzhou (southeast China), Fuzhou (southeast China), Lanzhou (northwest China), Wuhan (central China) and Shenyang (northeast China) (see Supplementary Fig. S1). One tertiary hospital was selected from each city. The enrolment eligibilities of the hospital were as follows: (1) accessible to every patient in the city, (2) had a neurology department for specialist-based clinical diagnosis of PD and Parkinsonism, (3) had an established computing system for medical records since 2003, and (4) willingness to participate in the study. The diagnosis of PD and Parkinsonism was made according to the “Chinese diagnostic criteria of Parkinson’s disease” (1999)^24^, which is essentially the same as UK Parkinson’s Disease Society Brain Bank Clinical Diagnostic Criteria (1992). Each comorbidity was diagnosed according to corresponding diagnostic criteria in China, which were essentially the same as the American or international diagnosis criteria. The data from all inpatients diagnosed with PD or Parkinsonism between January 2003 and December 2012 were collected. The exclusion criteria included the patients who were (1) under the age of 18 or (2) had insufficient clinical data. This study was approved by the Institutional Review Boards of Daping Hospital, Third Military Medical University. Obtainment of informed consent from participants was waived by the board due to the retrospective nature of the study, as per the Ethical Review of Biomedical Research Involving Human Beings issued by National Health and Family Planning Commission of China. All methods were performed in accordance with the approved guidelines and regulations.

### Data collection

Demographic and clinical data, including the name, age, age of diagnosis, gender, hospital registry number, admission/discharge date, total cost and all diagnoses, were collected from the computing system for medical records, which uses clinical codes from the 10^th^ International Classification of Diseases (ICD-10). Multiple hospitalizations were identified by the patient’s name, age, gender and registration number. The length of stay equalled the discharge date minus the admission date, and daily hospitalization expense (cost per day) was calculated by dividing the total cost by the length of stay.

### Comorbidity burden

ECI^25^ and CCI^26^, widely used to measure the disease burden, were applied to quantify the comorbidity burden in this study. The ECI consists of the following 30 comorbidities^25^: congestive heart failure, cardiac arrhythmias, valvular disease, pulmonary circulation disorders, peripheral vascular disorders, hypertension, paralysis, other neurological disorders, chronic pulmonary disease, uncomplicated diabetes, complicated diabetes, hypothyroidism, renal failure, liver disease, peptic ulcer disease without bleeding, AIDS, lymphoma, metastatic cancer, solid tumour without metastasis, rheumatoid arthritis, coagulopathy, obesity, weight loss, fluid and electrolyte disorders, blood loss anaemia, deficiency anaemia, alcohol abuse, drug abuse, psychoses and depression. The CCI consists of the following 17 comorbidities^26^: myocardial infarct, congestive heart failure, peripheral vascular disease, cerebrovascular disease, dementia, chronic pulmonary disease, connective tissue disease, ulcer disease, mild liver disease, diabetes, hemiplegia, moderate or severe renal disease, diabetes with end organ damage, any tumour, moderate or severe liver disease, metastatic solid tumour and AIDS. The comorbidities of included patients were collected to calculate the ECI and CCI.

### Statistical analysis

Characteristics of study subjects were described using percentages for categorical variables and means and standard deviations for continuous variables. The comorbidity burden, length of stay, and hospitalization expense were compared between PD and Parkinsonism patients and among different genders or age subgroups. For the between-group comparisons, continuous variables were compared using *t*-tests, and categorical variables were compared using a chi-square test. For the multi-group comparisons, measurement data from continuous variables were compared using ANOVA. The correlation between two variables was analysed with Spearman rank analysis. When the variances were not equal or the sample distribution did not conform to normality, nonparametric tests were used for analysis. A general linear model was applied to adjust the influence of age on the comorbidity profiles. All data entry processing was performed with EpiData software (Denmark), and the analysis was performed in SPSS. Statistical significance was defined as *P* < 0.05.

## Electronic supplementary material

Supplementary information
