# Supplementary material for: Comorbidity burden of patients with Parkinson’s disease and Parkinsonism between 2003 and 2012: A multicentre, nationwide, retrospective study in China
Source: Sci Rep. 2017 May 10;7:1671. doi: 10.1038/s41598-017-01795-0 (PMC5431825; doi:10.1038/s41598-017-01795-0)
Supplement: Supplementary file 1 — Supplementary information [file 41598_2017_1795_MOESM1_ESM.pdf]

Comorbidity burden of patients with Parkinson's disease and Parkinsonism between 2003 and 2012: A multicentre, nationwide, retrospective study in China

Xin Wang[1,+], Fan Zeng[1,+], Wang-Sheng Jin[1,+], Chi Zhu[1], Qing-Hua Wang[1], Xian-Le Bu[1], Hong-Bo Luo[2], Hai-Qiang Zou[3], Jie Pu[4], Zhong-He Zhou[5], Xiao-Ping Cui[6], Qing-Song Wang[7], Xiang-Qun Shi[2], Wei Han[8], Qiang Wu[4], Hui-Sheng Chen[5], Hang Lin[6], Li-Li Zhang[1], Meng Zhang[1], Yan Lian[1,9], Zhi-Qiang Xu[1], Hua-Dong Zhou[1], Tao Zhang[1,*], Yan-Jiang Wang[1]

Supplementary Information

Supplementary Figure S1.

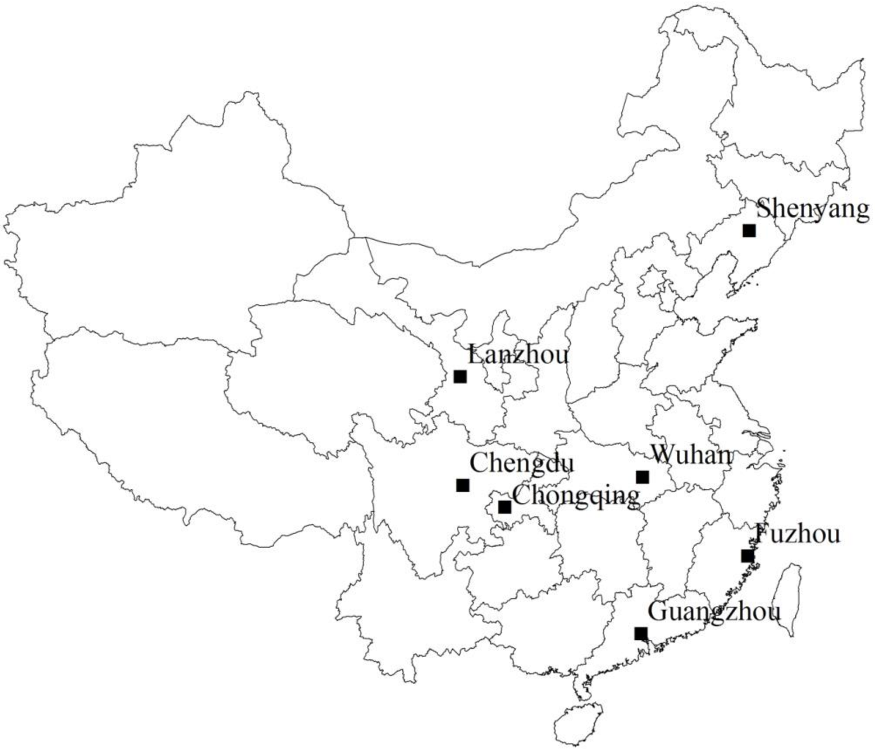

Supplementary Figure S1. Location of the seven cities selected in the study. The selected cities were Chongqing (southwest China), Chengdu (southwest China), Guangzhou (southeast China), Fuzhou (southeast China), Lanzhou (northwest China), Nanjing (east China), Wuhan (central China) and Shenyang (northeast China). This map was created using the ArcGIS10.0 software (Environmental Systems Research Institute, Inc).

Supplementary Table S1. The prevalence of concomitant disease included in ECI and CCI for all patients, PD patients, Parkinsonism patients

	Overall	PD	Parkinsonism	*P* value [a]
ECI	1.0(1.2)	1.0(1.2)	1.1(1.2)	
CCI	1.1(1.5)	1.1(1.5)	1.3(1.6)	
Myocardial infarction	43(1.03)	35(1.04)	8(0.97)	0.86
Congestive heart failure	113(2.70)	91(2.70)	22(2.67)	0.96
Peripheral vascular disease	93(2.22)	69(2.05)	24(2.92)	0.13
Cerebrovascular disease	1870(44.63)	1432(42.53)	438(53.22)	<0.001
Diabetes without chronic complication	453(10.81)	357(10.60)	96(11.66)	0.38
Chronic pulmonary disease	286(6.83)	235(6.98)	51(6.20)	0.43
Rheumatoid arthritis/collagen vascular	37(0.88)	31(0.92)	6(0.73)	0.60

diseases				
Peptic ulcer disease without bleeding	33(0.79)	27(0.80)	6(0.73)	0.83
Mild liver disease	192(4.58)	169(5.02)	23(2.79)	0.0062
Dementia	206(4.92)	148(4.40)	58(7.05)	0.0016
Paralysis	277(6.61)	186(5.52)	91(11.06)	<0.001
Renal failure	72(1.72)	54(1.60)	18(2.19)	0.25
Diabetes with chronic complication	63(1.50)	47(1.40)	16(1.94)	0.25
Solid tumor without metastasis	142(3.39)	126(3.74)	16(1.94)	0.011
Lymphoma	2(0.05)	2(0.06)	0(0.00)	1.00
Moderate or severe liver disease	15(0.36)	14(0.42)	1(0.12)	0.33
Metastatic cancer	44(1.05)	36(1.07)	8(0.97)	0.81
AIDS/HIV	1(0.02)	1(0.03)	0(0.00)	1.00
Cardiac arrhythmias	196(4.68)	153(4.54)	43(5.22)	0.41

Valvular disease	26(0.62)	22(0.65)	4(0.49)	0.58
Pulmonary circulation disorders	65(1.55)	49(1.46)	16(1.94)	0.31
Hypertension	1438(34.32)	1117(33.17)	321(39.00)	0.0016
Other neurological disorders	226(5.39)	177(5.26)	49(5.95)	0.43
Hypothyroidism	13(0.31)	12(0.36)	1(0.12)	0.48
Coagulopathy	5(0.12)	4(0.12)	1(0.12)	1.00
Obesity	6(0.14)	4(0.12)	2(0.24)	0.34
Weight loss	24(0.57)	15(0.45)	9(1.09)	0.037
Fluid and electrolyte disorders	91(2.17)	73(2.17)	18(2.19)	0.97
Blood loss anaemia	9(0.21)	7(0.21)	2(0.24)	0.69
Deficiency anaemia	84(2.00)	65(1.93)	19(2.31)	0.49
Alcohol abuse	10(0.24)	6(0.18)	4(0.49)	0.11
Drug abuse	18(0.43)	11(0.33)	7(0.85)	0.066

Depression	52(1.24)	44(1.31)	8(0.97)	0.44
Psychoses	139（3.32）	110(3.27)	29(3.52)	0.71

[a] *P* values of chi-square test between PD and Parkinsonism group.

Supplementary Table S2. The age-specific prevalence of concomitant disease included in ECI and CCI for all patients

Overall	＜50	50≤age＜59	60≤age＜69	70≤age＜79	≥80
ECI	0.29(0.58)	0.50(0.80)	0.78(0.94)	1.18(1.24)	1.79(1.51)
CCI	0.22(0.68)	0.56(0.96)	0.85(1.09)	1.33(1.48)	2.11(1.99)
Myocardial infarction	0(0.00)	0(0.00)	2(0.18)	17(1.15)	24(3.56)
Congestive heart failure	1(0.36)	1(0.16)	7(0.62)	46(3.12)	58(8.59)
Peripheral vascular disease	1(0.36)	3(0.48)	27(2.39)	43(2.91)	19(2.81)
Cerebrovascular disease	27(9.68)	182(28.98)	468(41.34)	784(53.12)	409(60.59)
Diabetes without chronic complication	4(1.43)	40(6.37)	98(8.66)	209(14.16)	102(15.11)
Chronic pulmonary disease	3(1.08)	5(0.80)	36(3.18)	121(8.20)	121(17.93)
Rheumatoid arthritis/collagen vascular diseases	1(0.36)	2(0.32)	17(1.50)	13(0.88)	4(0.59)

Peptic ulcer disease without bleeding	1(0.36)	2(0.32)	3(0.27)	11(0.75)	16(2.37)
Mild liver disease	7(2.51)	22(3.50)	53(4.68)	75(5.08)	35(5.19)
Dementia	1(0.36)	6(0.96)	16(1.41)	75(5.08)	108(16.00)
Paralysis	2(0.72)	12(1.91)	40(3.53)	117(7.93)	106(15.70)
Renal failure	1(0.36)	4(0.64)	7(0.62)	19(1.29)	41(6.07)
Diabetes with chronic complication	0(0.00)	4(0.64)	12(1.06)	29(1.96)	18(2.67)
Solid tumor without metastasis	1(0.36)	13(2.07)	26(2.30)	54(3.66)	48(7.11)
Lymphoma	0(0.00)	0(0.00)	0(0.00)	2(0.14)	0(0.00)
Moderate or severe liver disease	0(0.00)	3(0.48)	3(0.27)	4(0.27)	5(0.74)
Metastatic cancer	1(0.36)	2(0.32)	8(0.71)	19(1.29)	14(2.07)
AIDS/HIV	0(0.00)	0(0.00)	1(0.09)	0(0.00)	0(0.00)
Cardiac arrhythmias	1(0.36)	7(1.11)	26(2.30)	81(5.49)	81(12.00)
Valvular disease	1(0.36)	1(0.16)	8(0.71)	7(0.47)	9(1.33)

Pulmonary circulation disorders	0(0.00)	1(0.16)	13(1.15)	30(2.03)	21(3.11)
Hypertension	13(4.66)	110(17.52)	327(28.89)	626(42.41)	362(53.63)
Other neurological disorders	15(5.38)	24(3.82)	70(6.18)	88(5.96)	29(4.30)
Hypothyroidism	1(0.36)	1(0.16)	3(0.27)	3(0.20)	5(0.74)
Coagulopathy	0(0.00)	0(0.00)	1(0.09)	1(0.07)	3(0.44)
Obesity	0(0.00)	0(0.00)	1(0.09)	3(0.20)	2(0.30)
Weight loss	0(0.00)	2(0.32)	4(0.35)	10(0.68)	8(1.19)
Fluid and electrolyte disorders	3(1.08)	4(0.64)	12(1.06)	35(2.37)	37(5.48)
Blood loss anaemia	0(0.00)	1(0.16)	1(0.09)	4(0.27)	3(0.44)
Deficiency anaemia	3(1.08)	6(0.96)	6(0.53)	39(2.64)	30(4.44)
Alcohol abuse	1(0.36)	4(0.64)	2(0.18)	2(0.14)	1(0.15)
Drug abuse	0(0.00)	5(0.80)	8(0.71)	3(0.20)	2(0.30)
Depression	4(1.43)	7(1.11)	20(1.77)	12(0.81)	9(1.33)

Psychoses	15(5.38)	27(4.30)	41(3.62)	39(2.64)	17(2.52)

Supplementary Table S3. The age-specific prevalence of comorbidities included in ECI and CCI for PD patients

PD	＜50	50≤age＜59	60≤age＜69	70≤age＜79	≥80
ECI	0.29(0.59)	0.46(0.76)	0.77(0.94)	1.17(1.22)	1.81(1.53)
CCI	0.19(0.56)	0.54(0.92)	0.84(1.11)	1.29(1.44)	2.11(2.02)
Myocardial infarction	0(0.00)	0(0.00)	2(0.22)	13(1.13)	20(3.88)
Congestive heart failure	1(0.43)	1(0.19)	7(0.75)	35(3.04)	47(9.13)
Peripheral vascular disease	0(0.00)	1(0.19)	20(2.15)	33(2.86)	15(2.91)
Cerebrovascular disease	19(8.12)	155(28.92)	365(39.29)	594(51.52)	299(58.06)
Diabetes without chronic complication	3(1.28)	30(5.60)	83(8.93)	162(14.05)	79(15.34)
Chronic pulmonary disease	3(1.28)	4(0.75)	32(3.44)	101(8.76)	95(18.45)
Rheumatoid arthritis/collagen vascular diseases	1(0.43)	2(0.37)	14(1.51)	10(0.87)	4(0.78)

Peptic ulcer disease without bleeding	1(0.43)	2(0.37)	3(0.32)	9(0.78)	12(2.33)
Mild liver disease	7(2.99)	21(3.92)	49(5.27)	63(5.46)	30(5.83)
Dementia	1(0.43)	3(0.56)	12(1.29)	49(4.25)	83(16.12)
Paralysis	2(0.85)	8(1.49)	26(2.80)	77(6.68)	73(14.17)
Renal failure	1(0.43)	4(0.75)	5(0.54)	16(1.39)	28(5.44)
Diabetes with chronic complication	0(0.00)	4(0.75)	10(1.08)	19(1.65)	14(2.72)
Solid tumor without metastasis	1(0.43)	13(2.43)	24(2.58)	46(3.99)	42(8.16)
Lymphoma	0(0.00)	0(0.00)	0(0.00)	2(0.17)	0(0.00)
Moderate or severe liver disease	0(0.00)	3(0.56)	2(0.22)	4(0.35)	5(0.97)
Metastatic cancer	0(0.00)	1(0.19)	8(0.86)	15(1.30)	12(2.33)
AIDS/HIV	0(0.00)	0(0.00)	1(0.11)	0(0.00)	0(0.00)
Cardiac arrhythmias	0(0.00)	5(0.93)	21(2.26)	60(5.20)	67(13.01)
Valvular disease	1(0.43)	1(0.19)	7(0.75)	5(0.43)	8(1.55)

Pulmonary circulation disorders	0(0.00)	1(0.19)	12(1.29)	22(1.91)	14(2.72)
Hypertension	12(5.13)	92(17.16)	258(27.77)	485(42.06)	270(52.43)
Other neurological disorders	11(4.70)	18(3.36)	57(6.14)	73(6.33)	18(3.50)
Hypothyroidism	1(0.43)	0(0.00)	3(0.32)	3(0.26)	5(0.97)
Coagulopathy	0(0.00)	0(0.00)	1(0.11)	1(0.09)	2(0.39)
Obesity	0(0.00)	0(0.00)	0(0.00)	2(0.17)	2(0.39)
Weight loss	0(0.00)	1(0.19)	3(0.32)	6(0.52)	5(0.97)
fluid and electrolyte disorders	2(0.85)	4(0.75)	9(0.97)	26(2.25)	32(6.21)
Blood loss anaemia	0(0.00)	1(0.19)	1(0.11)	2(0.17)	3(0.58)
Deficiency anaemia	3(1.28)	5(0.93)	5(0.54)	26(2.25)	26(5.05)
Alcohol abuse	1(0.43)	2(0.37)	0(0.00)	2(0.17)	1(0.19)
Drug abuse	0(0.00)	1(0.19)	7(0.75)	2(0.17)	1(0.19)
Depression	3(1.28)	7(1.31)	16(1.72)	10(0.87)	8(1.55)

Psychoses	13(5.56)	17(3.17)	34(3.66)	34(2.95)	12(2.33)

Supplementary Table S4. The age-specific prevalence of comorbidities included in ECI and CCI for Parkinsonism patients

Parkinsonism	＜50	50≤age＜59	60≤age＜69	70≤age＜79	≥80
ECI	0.29(0.55)	0.71(0.98)	0.80(0.92)	1.22(1.31)	1.73(1.43)
CCI	0.36(1.11)	0.64(1.17)	0.90(1.00)	1.46(1.61)	2.10(1.89)
Myocardial infarction	0(0.00)	0(0.00)	0(0.00)	4(1.24)	4(2.50)
Congestive heart failure	0(0.00)	0(0.00)	0(0.00)	11(3.41)	11(6.88)
Peripheral vascular disease	1(2.22)	2(2.17)	7(3.45)	10(3.10)	4(2.50)
Cerebrovascular disease	8(17.78)	27(29.35)	103(50.74)	190(58.82)	110(68.75)
Diabetes without chronic complication	1(2.22)	10(10.87)	15(7.39)	47(14.55)	23(14.38)
Chronic pulmonary disease	0(0.00)	1(1.09)	4(1.97)	20(6.19)	26(16.25)
Rheumatoid arthritis/collagen vascular diseases	0(0.00)	0(0.00)	3(1.48)	3(0.93)	0(0.00)
Peptic ulcer disease without bleeding	0(0.00)	0(0.00)	0(0.00)	2(0.62)	4(2.50)
Mild liver disease	0(0.00)	2(2.17)	4(1.97)	12(3.72)	5(3.13)

Dementia	0(0.00)	3(3.26)	4(1.97)	26(8.05)	25(15.63)
Paralysis	0(0.00)	4(4.35)	14(6.90)	40(12.38)	33(20.63)
Renal failure	0(0.00)	0(0.00)	2(0.99)	3(0.93)	13(8.13)
Diabetes with chronic complication	0(0.00)	0(0.00)	2(0.99)	10(3.10)	4(2.50)
Solid tumor without metastasis	0(0.00)	0(0.00)	2(0.99)	8(2.48)	6(3.75)
Lymphoma	0(0.00)	0(0.00)	0(0.00)	0(0.00)	0(0.00)
Moderate or severe liver disease	0(0.00)	0(0.00)	1(0.49)	0(0.00)	0(0.00)
Metastatic cancer	1(2.22)	1(1.09)	0(0.00)	4(1.24)	2(1.25)
AIDS/HIV	0(0.00)	0(0.00)	0(0.00)	0(0.00)	0(0.00)
Cardiac arrhythmias	1(2.22)	2(2.17)	5(2.46)	21(6.50)	14(8.75)
Valvular disease	0(0.00)	0(0.00)	1(0.49)	2(0.62)	1(0.63)
Pulmonary circulation disorders	0(0.00)	0(0.00)	1(0.49)	8(2.48)	7(4.38)
Hypertension	1(2.22)	18(19.57)	69(33.99)	141(43.65)	92(57.50)

Other neurological disorders	4(8.89)	6(6.52)	13(6.40)	15(4.64)	11(6.88)
Hypothyroidism	0(0.00)	1(1.09)	0(0.00)	0(0.00)	0(0.00)
Coagulopathy	0(0.00)	0(0.00)	0(0.00)	0(0.00)	1(0.63)
Obesity	0(0.00)	0(0.00)	1(0.49)	1(0.31)	0(0.00)
Weight loss	0(0.00)	1(1.09)	1(0.49)	4(1.24)	3(1.88)
Fluid and electrolyte disorders	1(2.22)	0(0.00)	3(1.48)	9(2.79)	5(3.13)
Blood loss anaemia	0(0.00)	0(0.00)	0(0.00)	2(0.62)	0(0.00)
Deficiency anaemia	0(0.00)	1(1.09)	1(0.49)	13(4.02)	4(2.50)
Alcohol abuse	0(0.00)	2(2.17)	2(0.99)	0(0.00)	0(0.00)
Drug abuse	0(0.00)	4(4.35)	1(0.49)	1(0.31)	1(0.63)
Depression	1(2.22)	0(0.00)	4(1.97)	2(0.62)	1(0.63)
Psychoses	2(4.44)	10(10.87)	7(3.45)	5(1.55)	5(3.13)

Supplementary Table S5. Length of stay and hospitalization expense for PD patients

PD	Length of Stay(day)			Cost per Day [a]		
	Mean(SD)		*P* value	Mean(SD)		*P* value
Sex		Z=-4.135	<0.001		Z=-3.818	<0.001
Male	17.0(16.8)			1.09(1.04)		
Female	13.7(12.7)			0.94(0.69)		
Age		χ^2=134.786	<0.001		χ^2=19.159	=0.001
<50	11.1(10.7)			0.78(0.43)		
50-59	10.3(6.3)			0.97(0.71)		
60-69	13.9(15.6)			1.01(1.24)		
70-79	16.5(14.4)			1.07(0.77)		
≥80	23.9(20.5)			1.12(0.90)		

ECI		χ^2=89.447	<0.001		χ^2=45.851	<0.001
0	13.0(16.5)			0.91(1.10)		
1	16.3(15.6)			1.03(0.77)		
2	16.1(13.1)			1.12(0.87)		
3	20.1(15.2)			1.13(1.00)		
4	19.2(13.7)			1.35(0.78)		
5+	26.7(12.7)			1.21(0.62)		
CCI		χ^2= 101.833	<0.001		χ^2=53.992	<0.001
0	13.8(15.8)			0.88(0.67)		
1	14.4(14.7)			1.00(1.04)		
2	16.1(13.0)			1.14(0.75)		
3	19.9(15.3)			1.32(1.00)		
4	26.1(23.4)			1.32(1.71)		

5+	25.4(13.2)	1.12(0.62)

[a] Each value is divided by 1057 for standardization.

Supplementary Table S6. Length of stay and hospitalization expense for Parkinsonism patients

PD	Length of Stay(day)			Cost per Day [a]		
	Mean(SD)		*P* value	Mean(SD)		*P* value
Sex		Z=-0.786	=0.432		Z=-1.595	=0.111
Male	14.9(11.2)			1.20(0.81)		
Female	13.5(10.2)			1.10(1.09)		
Age		χ^2=6.567	=0.161		χ^2=14.151	=0.007
<50	18.1(14.5)			0.84(0.48)		
50-59	14.4(9.0)			0.73(0.36)		
60-69	11.0(4.9)			1.12(1.18)		
70-79	14.5(11.7)			1.27(0.83)		

≥80	19.1(14.3)			1.31(0.81)		
ECI		χ^2=8.395	=0.136		χ^2=21.362	=0.001
0	13.4(12.1)			0.88(0.54)		
1	13.5(10.3)			1.04(0.53)		
2	17.6(10.8)			1.46(1.01)		
3	13.0(7.6)			1.98(2.12)		
4	17.4(5.2)			1.97(1.34)		
5+	12.7(-)			1.41(-)		
CCI		χ^2= 13.829	=0.017		χ^2=35.791	<0.001
0	16.8(14.6)			0.73(0.50)		
1	11.5(6.7)			1.04(0.58)		
2	14.9(12.1)			1.67(1.31)		
3	19.3(12.7)			1.32(1.19)		

4	11.2(3.6)	1.43(0.61)
5+	19.9(5.5)	1.63(1.19)

[a] Each value is divided by 1057 for standardization.

Supplementary Table S7. The comorbidities profiles of different types of Parkinsonism

	Drug-induced secondary parkinsonism (n=9)	secondary parkinsonism due to other external agents (n=3)	postencephalitic parkinsonism (n=2)	vascular parkinsonism (n=330)	progressive supranuclear palsy (n=9)	multiple system atrophy (n=5)	other secondary parkinsonism (n=115)	secondary parkinsonism, unspecified (n=350)
Myocardial infarction	0 (0.0)	0 (0.0)	0 (0.0)	6 (1.8)	0 (0.0)	0 (0.0)	0 (0.0)	2 (0.6)
Congestive heart failure	0 (0.0)	0 (0.0)	0 (0.0)	10 (3.0)	0 (0.0)	0 (0.0)	4 (3.5)	8 (2.3)
Peripheral vascular	0 (0.0)	0 (0.0)	0 (0.0)	12 (3.6)	0 (0.0)	0 (0.0)	6 (5.2)	6 (1.7)

disease								
Cerebrovascular disease	1 (11.1)	0 (0.0)	2 (100.0)	278 (84.2)	7 (77.8)	0 (0.0)	60 (52.2)	46 (13.1)
Diabetes without chronic complication	0 (0.0)	0 (0.0)	1 (50.0)	56 (17.0)	2 (22.2)	1 (20.0)	14 (12.2)	23 (6.6)
Chronic pulmonary disease	0 (0.0)	0 (0.0)	0 (0.0)	35 (10.6)	1 (11.1)	0 (0.0)	4 (3.5)	12 (3.4)
Rheumatoid arthritis/collagen vascular diseases	0 (0.0)	0 (0.0)	0 (0.0)	1 (0.3)	0 (0.0)	0 (0.0)	0 (0.0)	5 (1.4)
Peptic ulcer disease without bleeding	0 (0.0)	0 (0.0)	0 (0.0)	4 (1.2)	0 (0.0)	0 (0.0)	1 (0.9)	1 (0.3)
Mild liver disease	1 (11.1)	0 (0.0)	0 (0.0)	11 (3.3)	0 (0.0)	0 (0.0)	8 (7.0)	3 (0.9)

Dementia	0 (0.0)	0 (0.0)	1 (50.0)	29 (8.8)	0 (0.0)	0 (0.0)	20 (17.4)	5 (1.4)
Paralysis	0 (0.0)	0 (0.0)	0 (0.0)	70 (21.2)	1 (11.1)	0 (0.0)	18 (15.7)	3 (0.9)
Renal failure	0 (0.0)	0 (0.0)	0 (0.0)	10 (3.0)	0 (0.0)	0 (0.0)	5 (4.3)	3 (0.9)
Diabetes with chronic complication	0 (0.0)	0 (0.0)	0 (0.0)	11 (3.3)	0 (0.0)	0 (0.0)	3 (2.6)	2 (0.6)
Solid tumor without metastasis	0 (0.0)	0 (0.0)	0 (0.0)	9 (2.7)	0 (0.0)	0 (0.0)	1 (0.9)	6 (1.7)
Lymphoma	0 (0.0)	0 (0.0)	0 (0.0)	0 (0.0)	0 (0.0)	0 (0.0)	0 (0.0)	0 (0.0)
Moderate or severe liver disease	0 (0.0)	0 (0.0)	0 (0.0)	1 (0.3)	0 (0.0)	0 (0.0)	0 (0.0)	0 (0.0)
Metastatic cancer	0 (0.0)	0 (0.0)	0 (0.0)	4 (1.2)	0 (0.0)	0 (0.0)	0 (0.0)	4 (1.1)
AIDS/HIV	0 (0.0)	0 (0.0)	0 (0.0)	0 (0.0)	0 (0.0)	0 (0.0)	0 (0.0)	0 (0.0)
Cardiac arrhythmias	0 (0.0)	0 (0.0)	1 (50.0)	17 (5.2)	1 (11.1)	0 (0.0)	11 (9.6)	13 (3.7)

Valvular disease	0 (0.0)	0 (0.0)	0 (0.0)	2 (0.6)	0 (0.0)	0 (0.0)	0 (0.0)	2 (0.6)
Pulmonary circulation disorders	0 (0.0)	0 (0.0)	0 (0.0)	9 (2.7)	0 (0.0)	0 (0.0)	3 (2.6)	4 (1.1)
Hypertension	0 (0.0)	0 (0.0)	1 (100.0)	200 (60.6)	3 (33.3)	1 (20.0)	50 (43.5)	66 (18.9)
Other neurological disorders	1 (11.1)	2 (66.7)	0 (0.0)	17 (5.2)	2 (22.2)	2 (40.0)	14 (12.2)	12 (3.4)
Hypothyroidism	1 (11.1)	0 (0.0)	0 (0.0)	0 (0.0)	0 (0.0)	0 (0.0)	0 (0.0)	0 (0.0)
Coagulopathy	0 (0.0)	0 (0.0)	0 (0.0)	0 (0.0)	0 (0.0)	0 (0.0)	0 (0.0)	1 (0.3)
Obesity	0 (0.0)	0 (0.0)	0 (0.0)	2 (0.6)	0 (0.0)	0 (0.0)	0 (0.0)	0 (0.0)
Weight loss	0 (0.0)	0 (0.0)	0 (0.0)	4 (1.2)	0 (0.0)	0 (0.0)	2 (1.7)	3 (0.9)
Fluid and electrolyte disorders	0 (0.0)	0 (0.0)	0 (0.0)	9 (2.7)	0 (0.0)	0 (0.0)	2 (1.7)	7 (2.0)
Blood loss anaemia	0 (0.0)	0 (0.0)	0 (0.0)	1 (0.3)	0 (0.0)	0 (0.0)	0 (0.0)	1 (0.3)

Deficiency anaemia	1 (11.1)	0 (0.0)	0 (0.0)	3 (0.9)	0 (0.0)	0 (0.0)	1 (0.9)	1 (0.3)
Alcohol abuse	4 (44.4)	0 (0.0)	0 (0.0)	0 (0.0)	0 (0.0)	0 (0.0)	0 (0.0)	0 (0.0)
Drug abuse	4 (44.4)	0 (0.0)	0 (0.0)	1 (0.3)	0 (0.0)	0 (0.0)	1 (0.9)	1 (0.3)
Depression	0 (0.0)	0 (0.0)	0 (0.0)	3 (0.9)	0 (0.0)	0 (0.0)	1 (0.9)	4 (1.1)
Psychoses	4 (44.4)	0 (0.0)	0 (0.0)	11 (3.3)	0 (0.0)	0 (0.0)	3 (2.6)	11 (3.1)
